# A New Diagnosis of Autoimmune Addison’s Disease in an 80-Year-Old Female

**DOI:** 10.7759/cureus.42201

**Published:** 2023-07-20

**Authors:** Pranjali Sharma

**Affiliations:** 1 Endocrinology, Parkview Medical Center, Pueblo, USA

**Keywords:** covid-19, geriatric, autoimmune, addison's disease, adrenal insufficiency, hyponatremia

## Abstract

Hyponatremia is a common cause of hospitalization in older adults. Addison’s disease (AD), an uncommon cause of hyponatremia, is primary adrenal insufficiency (AI) caused by autoimmune adrenalitis, infections, adrenal hemorrhage, or infiltration. Autoimmune adrenalitis is an uncommon cause of AD after age 60 years. We report the case of an 80-year-old female with steroid-controlled asthma, who was diagnosed with primary AI after presenting with weakness, nausea, vomiting, and hyponatremia two weeks after a urinary tract infection (UTI). Further testing revealed a positive 21 hydroxylase antibody confirming autoimmune AD. The patient has done well on hydrocortisone and fludrocortisone daily with occasional need for stress dosing during infections. AD should be considered as a cause of hyponatremia in hospitalized elderly patients. Non-specificity of symptoms of AD makes the diagnosis difficult in this age group, often causing delays in the appropriate management. Our patient's autoimmune AD was masked by intermittent steroid use over the years. During the COVID-19 pandemic, masking and social distancing decreased her steroid needs and unmasked AD after the UTI. We highlight the importance of considering AD as a cause of hyponatremia and autoimmune AD as a cause of AI even in the elderly.

## Introduction

Hyponatremia is a common cause of hospitalization in older adults. Severe hyponatremia is associated with high mortality [1]. Common causes of hyponatremia among the elderly include medications, syndrome of inappropriate antidiuretic hormone secretion (SIADH), and conditions such as diabetes mellitus (DM), heart failure, malignancies, or infections [2]. While endocrinopathies such as hypopituitarism and adrenal insufficiency (AI) are less common causes of hyponatremia, hyponatremia is the most common electrolyte disorder in AI and hypopituitarism [1]. In fact, AI should be ruled out as a cause of hyponatremia, when making a diagnosis of idiopathic SIADH.

AI can be primary or secondary. Primary AI or Addison's disease (AD) is the inadequate production of adrenocorticosteroids due to adrenal gland damage. Common etiologies include autoimmune adrenalitis, infection, tumor, or hemorrhage [3]. The incidence of AD is 0.6 per 100,000 of the population per year and the common age of presentation is between 30 and 50 years in adults [4]. AD tends to be rarer in the older population and the exact prevalence is not clear. Furthermore, aging leads to higher cortisol levels and diminished hypothalamic-pituitary-adrenal (HPA) axis sensitivity, along with diminished immunity, which makes autoimmune AD even rarer in the elderly [5]. We report the case of an 80-year-old female who was hospitalized with hyponatremia and ultimately diagnosed with autoimmune AD.

This case report was presented as a poster presentation at ENDO 2022 in Atlanta, Georgia, United States.

## Case presentation

An 80-year-old female with a previous medical history of asthma and type 2 DM, presented to the emergency room with weakness, imbalance, nausea, and vomiting that began two weeks after a urinary tract infection (UTI) treated with an oral antibiotic. She was on insulin and metformin at the time of presentation.

Diagnostic assessment

The patient's blood pressure was 96/64 mm Hg, pulse was 98 beats/minute, and regular and temperature was 98.8F (Table 1). While the patient was lethargic, she was oriented to time, place, and self. General examination was unremarkable with the exception of mild generalized abdominal pain attributed to frequent vomiting.

**Table 1:** Vital signs during the course of the patient’s illness

Vital signs	Inpatient				Outpatient
	Day 1	Day 4	Day 6	Day 9	Day 30
Blood Pressure	96/64	126/61	104/46	98/59	102/60
Heart Rate	98	100	86	95	76
Temperature	98.8	97.1	99 F	98.6 F	97.9

Laboratory evaluation revealed hyponatremia (sodium 120 mmol/L, normal 134-145 mmol/L), hyperkalemia (potassium 5.7 mmol/L, normal 3.5-5.1 mmol/L, hypochloremia (chloride 91 mmol/L, normal 98-107 mmol/L) with acute kidney injury (creatinine 1.2 mg/dL, normal 0.55-1.02 mg/dL, glomerular filtration rate (GFR) 43 mL/min/1.73m^2^, normal >=60 mL/min/1.73m^2^) (Table 2). A complete blood count revealed eosinophilia (1.44 10-3/uL, normal 0.04-0.54 10-3/uL) but no signs of an infection (Table 2). Glycosylated hemoglobin (HbA1c) was 6.1%. Serum osmolality or urine studies were not done. A previous thyroid testing panel had revealed normal thyroid function a few months prior.

**Table 2:** Laboratory values during the course of the patient's illness GFR: glomerular filtration rate

Laboratory results	Inpatient					Outpatient
	Normal	Day 1	Day 4	Day 6	Day 9	Day 30
Sodium (mmol/L)	134-145	120	124	123	126	139
Potassium (mmol/L)	3.5-5.1	5.7	4.7	4.3	4.3	4.6
Chloride (mmol/L)	98-107	91	97	101	96	101
Creatinine (mg/dL)	0.55-1.02	1.2	0.77	0.60	0.57	0.76
GFR (ml/min/1.73 m^2^)	>=60	43	72	96	102	75
Eosinophils (10-3/ul)	0.04-0.54	1.44	1.35	0.87	1.06	0.73
Serum osmolality (mosm/kg)	278-298				271	288

Treatment

The patient was diagnosed with dehydration due to vomiting and was admitted for treatment with intravenous normal saline, insulin, and dextrose therapy for acute kidney injury, hyponatremia, and hyperkalemia. Hyperkalemia improved by day 4 (potassium 4.7 mmol/L), however, hyponatremia persisted (Na 123-126 mmol/L) for up to a week into admission (Table 2). The patient was placed on salt tablets, free water restriction, and ongoing intravenous normal saline. By day 9, only hyponatremia (sodium 126 mmol/L) and eosinophilia (1.06 10-3/uL) persisted (Table 2). The patient also remained hypotensive with blood pressure readings ranging between 90/40 and 120/60 mm Hg (Table 1).

Other causes for hyponatremia were sought due to lack of improvement. On further questioning, the patient reported several decades of intermittent prednisone use for asthma exacerbations for periods lasting between days and weeks. Iatrogenic secondary adrenal insufficiency as a cause of hyponatremia was considered and a fasting cortisol was noted to be low (4.63 mcg/dL) (Table 3). A 250 mcg cosyntropin stimulation test was conducted with baseline adrenocorticotropic hormone (ACTH) levels. The testing revealed an elevated ACTH level (252 pg/mL, normal 7.2-63.3 pg/mL) and poor cortisol response (baseline 4.5 mcg/dL --> 8 mcg/dL at 60 minutes) (Table 3). This suggested primary AI and the patient was placed on oral hydrocortisone 15 mg and fludrocortisone 0.1 mg daily. Thereafter, the patient's hyponatremia, blood pressure, and eosinophilia all improved, and she was discharged home (Tables 1-2). An outpatient endocrinology consultation was set up.

**Table 3:** Adrenal laboratory testing during the course of the patient's illness ACTH: adrenocorticotropic hormone

Adrenal laboratory results			
	Normal	Day 6	
AM Cortisol (mcg/dL)		4.63	
ACTH (pg/mL)	7.2-63.3	252	
Cosyntropin Stimulation Test (mcg/dL)			
	Inpatient		Outpatient
Cortisol Baseline	4.5		8.5
Cortisol, 30 mins	6.7		9.6
Cortisol, 60mins	8		10
21-Hydroxylase Antibody			Positive

At the endocrinology consultation (day 30 post initial emergency room (ER) visit), the patient reported having had worsening nausea and vomiting for at least 18 months prior to the hospitalization. Additionally, she had a weight loss of 50 lbs, weakness, and short-term memory loss which were attributed to poor dietary intake, and dizziness attributed to vertigo. She recalled that her symptoms got worse after the UTI. On examination, the patient did not have typical hyperpigmentation of AD. Her vitals showed blood pressure 102/60 mm/Hg, pulse 76/min, and temperature 97.9F (Table 1). The repeat cosyntropin stimulation test was still abnormal (baseline cortisol 8.5 mcg/dL--> 10 mcg/dL at 60 minutes) (Table 3). Computed tomography (CT) of the abdomen did not reveal an adrenal pathology. Surprisingly, 21-hydroxylase antibody testing done for the rare possibility of autoimmune AD was positive (Table 3).

Outcome and follow-up

Based on elevated ACTH, abnormal cosyntropin stimulation test results, and positive 21 hydroxylase antibody testing, the patient was given a confirmed diagnosis of autoimmune AD. She remains on combined glucocorticoid and mineralocorticoid replacement with hydrocortisone 15 mg in AM + 5 mg in PM and fludrocortisone 0.1 mg daily. She is now two years out from the diagnosis and has been doing well on this regimen with appropriate stress dosing during infections. Further testing for parathyroid gland function revealed normal parathyroid hormone level (22 pg/mL, reference 15-65 pg/mL) with normal calcium levels (8.9 mg/dL, reference 8.7-10.3 mg/dL). This ruled out the possibility of autoimmune polyglandular syndrome type 2 (APS type 2).

## Discussion

Hyponatremia is the most frequent electrolyte abnormality in elderly patients in the hospital and the community [2]. While age is an independent risk factor, impaired water-excretory capacity, exposure to medications (thiazides, selective serotonin reuptake inhibitors (SSRIs), serotonin and norepinephrine reuptake inhibitors (SNRIs), non-steroidal anti-inflammatory drugs (NSAIDs)), presence of diseases associated with hyponatremia (DM, infections, heart failure, endocrinopathies) and a "tea and toast" diet are other mechanisms leading to hyponatremia in the elderly [2]. Among endocrinopathies, idiopathic SIADH is one of the more frequent causes of hyponatremia in the elderly. The clinical manifestations of hyponatremia from SIADH resemble symptoms of AI. Moreover, hyponatremia is the most common electrolyte abnormality in AI [1]. Therefore, it is important to exclude AI in the evaluation of hyponatremia. Our elderly hyponatremic patient was not on contributing medications, neither did she have an ongoing infection. While diabetes could have been a contributing factor, her glycemic control was good enough to rule this out as the main cause. Thus, rarer causes of hyponatremia were checked.

AI is difficult to diagnose in the elderly due to the non-specificity of symptoms. Symptoms such as lethargy, asthenia, poor appetite, or weight loss can be mistaken for the aging process, especially when insidious [1,3]. Aging leads to a decline in the HPA axis sensitivity, causing decreased negative feedback on the secretion of cortisol. Mean cortisol levels increase, with irregular secretory patterns and a flattened circadian response, leading to an earlier attenuated morning cortisol peak and higher nighttime cortisol levels [5]. These changes contribute to features of frailty in the elderly population, such as weight loss, muscle wasting, and anorexia [5]. Our patient had an insidious onset of nausea, vomiting, weight loss, and weakness for 18 months prior to her ER presentation. However, this did not raise suspicion for AI until her hospitalization with severe hyponatremia and instead, was thought to be age-related.

Primary AI or AD is common in the third to fifth decade of life [3,4]. Secondary AI due to long-term steroid use for comorbidities such as asthma, chronic obstructive pulmonary disease (COPD), or degenerative joint disease, is more common in the later decades. When our patient reported that she used prednisone for asthma, secondary AI due to exogenous steroid use was considered as the main differential diagnosis. However, the patient had elevated ACTH levels, a characteristic feature of AD.

AD occurs due to a primary adrenal pathology such as an autoimmune, infectious, metastatic, or hemorrhagic destruction of the adrenal gland [3]. Autoimmune destruction of the adrenal gland cortex, caused by 21 hydroxylase antibodies, is the most common cause of AD. Aging compromises the normal functioning of the immune system through mechanisms like suppressor T cell augmentation, decreased B-cell antibody production, and reduced number of naive T cells. While this can be extrapolated to an age-related decrease in titer of 21 hydroxylase antibodies, a recent study of 711 patients with autoimmune AD suggested that these antibodies are detectable in patients more than 30 years after the initial diagnosis [6]. Therefore, 21 hydroxylase antibodies are good biomarkers for the diagnosis of autoimmune AD and should be tested even in the elderly population. We may diagnose a new case of autoimmune AD based on detectable antibodies, just like in our patient.

The diagnosis of AD based on low cortisol, elevated ACTH, and poor response to cosyntropin stimulation test, remains the same irrespective of age of diagnosis. In fact, elevated ACTH and low cortisol noted despite the normal aging-related HPA axis changes, are all the more characteristic of AD in the elderly. While other causes of AD are rare, it is important to obtain imaging of the adrenal glands, such as a CT scan, to evaluate for possible infections, cancer, or bleeding (particularly with concurrent anticoagulation).

Management of AD comprises lifelong glucocorticoid and mineralocorticoid supplementation. Glucocorticoid supplementation is provided through hydrocortisone 15-25 mg/day in 2-3 divided doses or an equivalent dose of prednisone. The dose is adjusted based on body weight, blood pressure, energy, and signs of steroid excess. ACTH levels should not be used to adjust dosing. Mineralocorticoid supplementation is through fludrocortisone 0.05-2 mg daily. The dose is adjusted to maintain blood pressure, electrolytes, and renin levels within the reference range [7]. During an illness, stress dosing with 2-3 times the maintenance dose of glucocorticoids is critical in AD to avoid adrenal crises.

Lastly, we would like to point out that the COVID-19 pandemic played a role in this patient's autoimmune AD diagnosis. This patient had multiple asthma exacerbations throughout her life, which she treated with prednisone. We believe that the intermittent steroid use masked autoimmune AD. With the advent of social distancing and masking mandates during the COVID-19 pandemic, she did not have asthma exacerbations as frequently, which led to decreased prednisone use. This unmasked the symptoms of AI that were aggravated after her UTI leading to the diagnosis of autoimmune AD.

## Conclusions

Based on our case report, we conclude that AD, while an uncommon cause of hyponatremia, should be considered in a hospitalized hyponatremic elderly patient. The non-specificity of symptomatology of AD in the elderly can make the diagnosis difficult but should be considered nonetheless. While autoimmune AD, characterized by 21-hydroxylase antibody-mediated destruction of the adrenal cortex, is uncommon in the elderly, it should be evaluated in this population too.
